# The Eye: A Possible New Route of Infection in COVID-19

**DOI:** 10.1017/dmp.2020.270

**Published:** 2020-07-27

**Authors:** Anis Abobaker, Aboubaker Alzwi

**Affiliations:** General Medicine, Spire Fylde Coast Hospital, Blackpool, UK; Internal Medicine, 7th of October Hospital, Benghazi, Libya

**Keywords:** COVID-19, routes of transmission, nasolacrimal drainage

The main routes of transmission of infection in coronavirus disease (COVID-19) are by respiratory droplets and direct contact. Recently, the possibility of transmission of infection through the eye has been considered. In fact, the close anatomical link between the eye and the respiratory tract enables viruses to be drained down through the nasolacrimal duct to the upper respiratory tract and cause infection.^1^ Interestingly, the epithelial receptors of ocular and respiratory tissue share a similar structure that might explain the ocular tropism of the respiratory viruses.^1^ Although ocular diseases have been commonly reported with principal respiratory viruses, such as adenovirus, influenza virus, respiratory syncytial virus, and rhinovirus, they are rarely reported with coronaviruses (CoVs).^1^ This might indicate that the eye is not a potential target for coronaviruses; however, this does not rule out the possibility that the conjunctiva and the ocular mucous membrane could act as a port of entry to CoVs to the upper respiratory tract given the close anatomical proximity and the similar epithelial receptors.

There is evidence that lack of eye protection in clinical settings increased the risk of infection of the severe acute respiratory disease (SARS) caused by SARS-CoV. During the outbreak of SARS in 2003, the inappropriate use of eye protection increased the risk of the transmission of SARS-CoV infection in Toronto, Canada.^1^ Furthermore, a member of the national expert panel on pneumonia contracted COVID-19 infection following an inspection visit to one of the hospitals in Wuhan city, despite using appropriate personal protective equipment (PPE), including a full gown and N95 face mask, but he did not wear goggles.^2^ In one experiment, rhesus macaques acquired COVID-19 infection through conjunctival inoculation.^3^ SARS-CoV-2 (virus) was detected in the conjunctival swab on day 1 post-inoculation, but when repeated after a few days, the polymerase chain reaction (PCR) result was negative.^3^ These findings further support the concept that CoVs transmit through the lacrimal drainage to the respiratory system, which explain why no virus is detected in the second conjunctival swab. In fact, all these observations put a great emphasis on the importance of using appropriate eye protection to prevent transmission of infection between patients and health care professionals. In addition, wearing protective eye glasses or face shields could be a mandatory requirement, particularly in closed public places where social distancing cannot be maintained, as most countries across the world are taking preparatory steps to implement partial or full release of lockdown.

The binding of SARS-CoV-2 viral membrane spike proteins with angiotensin converting enzyme 2 (ACE2) receptors facilitates viral cell entry and replication. This is considered as the main pathogenesis of COVID-19. Therefore, cells that express ACE2 receptors could be potential pathological targets for SARS-CoV-2. Variable rates of expression of ACE2 receptors have been detected in different ocular tissues, including the retina, cornea and conjunctiva, and the strongest expression level was detected in the cornea.^4-6^ This might indicate that the cornea could be targeted by SARS-CoV-2. So far, there is only 1 case report in the literature demonstrating corneal involvement in COVID-19 in a patient who presented with keratoconjunctivitis as the only clinical feature.^7^ Another case series study reported conjunctivitis as the initial and sole clinical presentation in 5 patients with confirmed COVID-19.^8^ Although it is rarely reported, eye disease, such as conjunctivitis, seems to be a recognized clinical feature of COVID-19. The low frequency of conjunctivitis and corneal involvement in COVID-19 patients could be explained with the fact that the level of ACE2 expression in ocular tissues is much less compared with other organs, such as the lungs and kidneys.^6^ Interestingly, conjunctivitis has been reported with a higher frequency in patients with severe COVID-19 compared with non-severe COVID-19, 3% vs 0.7%, respectively.^9^ This could be because patients with severe COVID-19 disease tend to have multi-organ involvement. Therefore, conjunctivitis might indicate poor outcome in COVID-19 patients with respiratory illness.

SARS-CoV-2 has been detected by PCR in conjunctival swabs taken from COVID-19 patients with conjunctivitis as well as patients without ocular manifestations.^10,11^ This might suggest that SARS-CoV-2 can be transmitted by tears. As the number of positive PCR results from ocular surface swabs is extremely low, larger numbers of patients are yet to be tested before confirming this possible new route of infection. Nonetheless, doctors working in the front lines should remain vigilant and wear complete and appropriate PPE when examining patients with suspected COVID-19, even if the patients do not have clinical features of eye diseases.
